# Supplementary material for: spVelo: RNA velocity inference for multi-batch spatial transcriptomics data
Source: Genome Biol. 2025 Aug 11;26:239. doi: 10.1186/s13059-025-03701-8 (PMC12337411; doi:10.1186/s13059-025-03701-8)
Supplement: Supplementary file 1 — Additional file 1. Contains supplementary texts, tables and figures. [file 13059_2025_3701_MOESM1_ESM.pdf]

1 Supplementary texts

Text S1: Ablation tests

To separate the contributions between different modules of spVelo, we conducted the following ablation tests:

1. Removing MMD penalty: To quantitatively show the contributions of spVelo's MMD penalty to velocity coherence across batches, we performed ablation studies by removing the MMD penalty. Then we compared the ablated model with the original spVelo using the cosine similarity score between the RNA velocities of different batches. Our results are shown in Fig. S6. According to this figure, spVelo has higher velocity coherence across batches than spVelo without MMD. We further performed an independent t-test between the two models, and the result shows that using the MMD penalty significantly improves inter-batch velocity coherence.
2. Replacing GAT module with GCN or GraphSAGE: We performed ablation studies to evaluate the choice of the GAT module compared to other graph architectures, specifically GCN and GraphSAGE, using the 9 benchmarking scores. The results are visualized in Fig. S12. From the boxplots in Fig. S12(a), in the simulated Pancreas dataset, spVelo outperforms the other ablated models, especially in the direction scores. On the other hand, in the OSCC dataset visualized in Fig. S12(b), spVelo performs comparable to spVelo with GCN module and better than spVelo with GraphSAGE module. As a result, we chose the GAT module for spVelo, based on these findings across both datasets.
3. Spatial and MNN edge weightings tuning: We further performed edge weightings tuning for spVelo. spVelo has equal weights for spatial and mnn edges. To explore the impact of this choice, we ran two other ablated models with spatial_weight:mnn_weight as 1:2 and 2:1, and named them as sp_1_mnn_2 and sp_2_mnn_1, respectively. The results are visualized as boxplots in Fig. S13. According to the figure, spVelo performs best in the simulated Pancreas dataset and performs comparably to the other models in the OSCC dataset. These results support the decision to set the same weights for spatial and mnn edges in spVelo.
4. Dirichlet prior distribution parameters: We performed ablation studies on the choice of the Dirichlet distribution parameters. The current Dirichlet prior distribution parameters for each state (induction, induction steady, repression, repression steady) is $(0.25, 0.25, 0.25, 0.25)$. We then include a sparser distribution with parameters $(0.1, 0.1, 0.1, 0.1)$, a middle distribution with parameters $(0.3, 0.3, 0.3, 0.3)$ and a denser distribution with parameters $(0.5, 0.5, 0.5, 0.5)$. We further include a steady-favored setting with parameters $(0.1, 0.5, 0.1, 0.5)$ and an active-favored setting with parameters $(0.5, 0.1, 0.5, 0.1)$. Results are visualized in Fig. S14. According to the figure, spVelo performs comparably to sparser, mid and denser settings, proving that the model performance is not sensitive to the choice of parameters. Steady-favored setting performs better in expr and mnn scores of the simulated Pancreas dataset, while both steady-favored setting and active-favored setting perform worse in the OSCC dataset. This may prove that the cells in the simulated Pancreas dataset have higher probability in the steady state. The pancreas dataset

is collected from E15.5, which is a steady phase for the full pancreatic development. On the other hand, there may be no preference in the OSCC dataset. As a result, we encourage users to assign data-specific parameters if they have clear evidence that the parameter setting is reasonable.

5. R-square threshold for filtering uninformative genes: We observed that filtering some of the MURK genes is beneficial to the performance of spVelo. We filtered genes based on their R-square from fitting a linear regression model on unspliced to spliced mRNA levels (i.e. running steady-state model of scVelo, R-square scores are extracted from .var.velocity_r2). Then we set the threshold as $0, 0.1, \ldots, 0.9$, and filtered genes with R-square score below this threshold. Results are visualized in Fig. S17. According to the figure, the best R-square threshold for filtering genes in the OSCC dataset is 0.2. This threshold effectively balances the inclusion of informative genes while excluding those with poor model fits, which was chosen for the implementation of spVelo.

Text S2: Benchmark of more methods and datasets

Despite differences in resolution and platform design, spVelo remains consistent across spatial datasets. This is due to spVelo's GAT module that can robustly encode spatial structures for estimating RNA velocity. To prove this, we benchmarked all methods on a new stereo-seq mousebrain dataset [1], processed with bin size 60. Since this dataset only has one batch, we only benchmarked the per-batch scores (i.e. expr and spatial scores). The cerebral cortex plays a central role in brain development and function, making it a particularly important region for studying cellular dynamics. For these reasons, we focused our benchmarking on Isocortex layers L2/3, L4, L5, and L6 of the mousebrain dataset. The results after minmax-scaling are summarized in Table S1. From the table, spVelo outperforms other methods, indicating its capability of accurately capturing the underlying cellular dynamics and its robustness on newer platforms.

To compare spVelo with existing spatially-aware velocity inference methods, we include our comparison between spVelo, STT[2], SIRV[3], scGen+STT, scGen+SIRV in Table S2. Same as the main text, by STT and SIRV, we refer to running them on each batch; by scGen+STT and scGen+SIRV, we refer to first using scGen to correct batches and then run STT and SIRV on the entire dataset. There are only per-batch scores (expr scores and spatial scores) for STT and SIRV since they are not run on the entire dataset. The final scores are calculated by averaging all batches and all seeds. Since SIRV requires paired scRNA-seq of the same tissue, we only benchmarked it using the original scRNA-seq Pancreas dataset and simulated spatial Pancreas dataset. According to Table S2, spVelo outperforms both per-batch mode and scGen mode of STT and SIRV. Therefore, we conclude that spVelo exhibits great advantages in the RNA velocity estimation task of spatial transcriptomics.

Text S3: Discussion of metrics

While the transition score and direction score may seem similar, they measure different aspects and serve as a complement to each other. The transition score is a direct assessment of how well the inferred velocity predicts changes in gene activity. However, the gene expression space is often high-dimensional and noisy. The results might be sensitive to noise or irrelevant features. On the other hand, the direction score measures the cosine similarity between the inferred velocity and the change in a low-dimension PCA space. This focuses on more general trends, yet is unable to reflect subtle but important biological information. As a result, using the two scores simultaneously can provide a more complete picture of how well the inferred velocity aligns with true biological processes and offers complementary insights into the performance of velocity inference.

In the dimension reduction process of direction score calculation, we used Principal Component Analysis (PCA) [4] instead of Uniform Manifold Approximation and Projection (UMAP) [5] that was used in [6, 7]. The reason for our choice is: Firstly, PCA has a higher dimension than UMAP, thus preserving more information from the original dataset. Secondly, PCA is deterministic while UMAP is stochastic, so PCA can provide more consistent results. To this end, we chose PCA over UMAP for direction score calculation.

Text S4: Extra analysis of data simulation

Other than scCube [8], we also considered scDesign3 [9] for simulating spatial datasets. In our implementation, we used sample 2 of the OSCC dataset [10] and followed the step-by-step tutorial of scDesign3 to simulate a new spatial dataset. However, the simulated dataset exhibits unreasonable expression data, as shown in the scatter plots in Fig. S16. The relationship between spliced expression levels and unspliced expression levels is incorrect.

Text S5: Scalability of spVelo

To prove the scalability of spVelo on larger datasets, we simulated using the scRNA-seq Pancreas dataset [11] and implemented the following two conditions:

1. large #slices with normal #cells per slice: we chose the number of slices to be $5, 10, 15, 20$ and each batch has $\sim 2,300$ cells.
2. normal #slices while large #cells per slice: we first simulated scRNA-seq with cell numbers $1\times$, $3\times$ and $6\times$ the size of the original dataset. Then we simulated the spatial transcriptomics from the simulated scRNA-seq. Here we chose the number of slices to be 3 for all settings.

The time and memory costs of all settings are reported in Fig. S18. From the figure, the changes of time and memory cost with different settings are almost linear. Even in the most demanding settings like 20 batches$\times \sim 2,300$ cells and 3 batches$\times \sim 13,000$

cells, spVelo estimates RNA velocity in under 1 hour and requires less than 50 GB of memory. This demonstrates the efficiency and scalability of spVelo, and we deduce that the GAT architecture doesn't introduce significant computational bottlenecks in our implementation. This demonstrates the efficiency of spVelo and we deduce that the GAT architecture doesn't introduce much computational bottleneck.

2 Supplementary tables

**method**	**expr**			**spatial**			**mean**
	confidence	transition	direction	confidence	transition	direction	
scVelo (stochastic)	0.583	0.442	0.261	0.574	0.067	0.622	0.425
scVelo (dynamical)	0.073	0.624	0.378	0	**1**	0	0.346
veloVI	0.401	0.458	0.459	0.492	0.605	0.027	0.407
LatentVelo (standard)	0.927	0	0	0.919	0.625	0.029	0.417
LatentVelo (annotated)	**1**	0.908	0.923	**1**	0	**1**	0.805
STT	0	0.286	0.132	0.235	0.350	0.062	0.177
**spVelo**	0.683	**1**	**1**	0.741	0.980	0.886	**0.882**

Table S1: Comparison between all methods on stereo-seq mousebrain dataset. The results are minmax-scaled. Bold values indicate the best performance in each column.

(a)

**method**	**expr**			**spatial**			**mnn**		
	confidence	transition	direction	confidence	transition	direction	confidence	transition	direction
scGen+STT	0.708	-3.788e-2	-0.045	0.345	-0.038	-0.090	0.689	-0.017	-0.003
scGen+SIRV	0.941	**-2.152e-3**	**0.024**	0.667	**-0.009**	-0.012	**0.915**	-0.003	-0.006
STT	0.841	-2.611e-3	0.007	0.347	-0.027	-0.067	-	-	-
SIRV	**0.970**	-2.482e-3	0.0225	**0.713**	-0.011	-0.012	-	-	-
**spVelo**	0.909	-3.491e-3	0.023	0.618	-0.014	**0.027**	0.855	**-0.0004**	**0.005**

(b)

**method**	**expr**			**spatial**			**mnn**		
	confidence	transition	direction	confidence	transition	direction	confidence	transition	direction
scGen+STT	0.300	2.609e-4	3.002e-4	0.343	8.437e-4	0.013	0.667	-4.755e-3	-2.291e-3
STT	0.743	**2.661e-3**	0.184	0.577	0.012	**0.226**	-	-	-
**spVelo**	**0.949**	1.261e-3	**0.189**	**0.933**	**0.015**	0.227	**0.757**	**-8.042e-5**	**0.031**

Table S2: Comparison of confidence, transition, and direction scores across spVelo, STT, SIRV, scGen+STT, scGen+SIRV. Bold values indicate the best performance in each column. (a) Comparison in the simulated Pancreas dataset. (b) Comparison in the OSCC dataset.

3 Supplementary figures

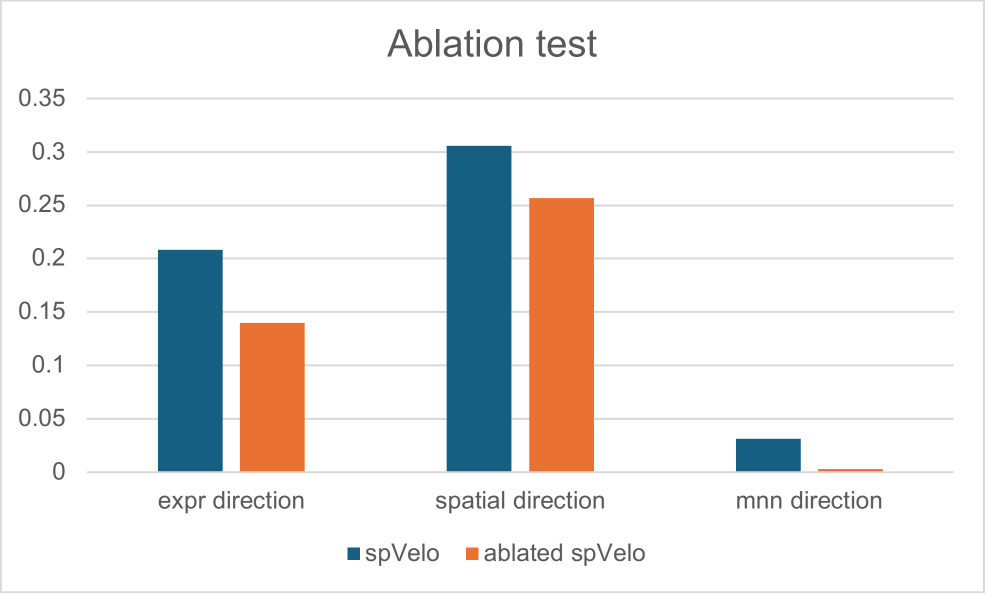

Fig. S1: Ablation test of spVelo in OSCC dataset, comparing expression direction, spatial expression, and mnn direction score between spVelo and spVelo without inputting spatial information.

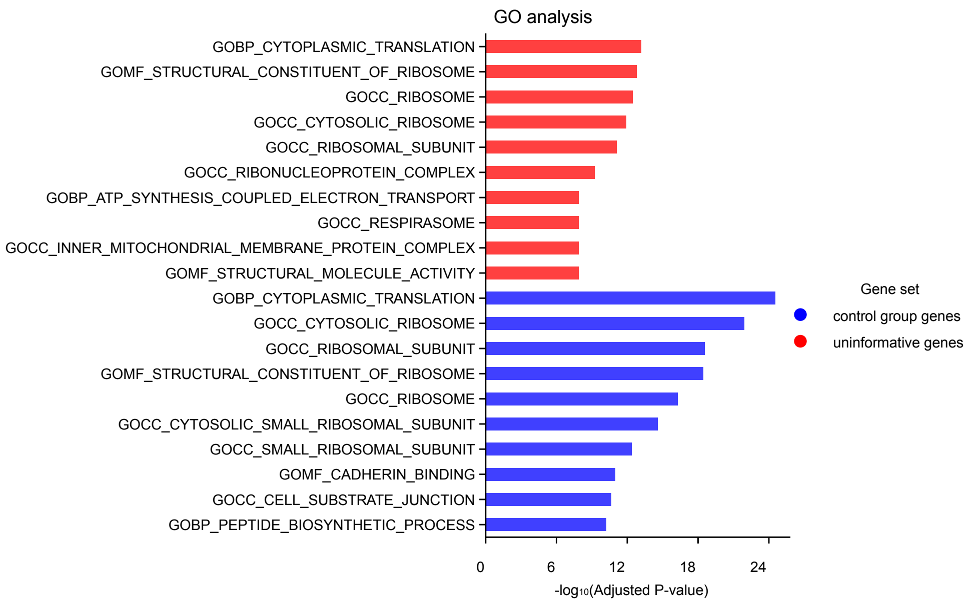

Fig. S2: Dotplot of GO analysis for comparing filtered uninformative genes with control group genes. The control group is randomly selected from informative genes, with the same number of genes as in the uninformative genes.

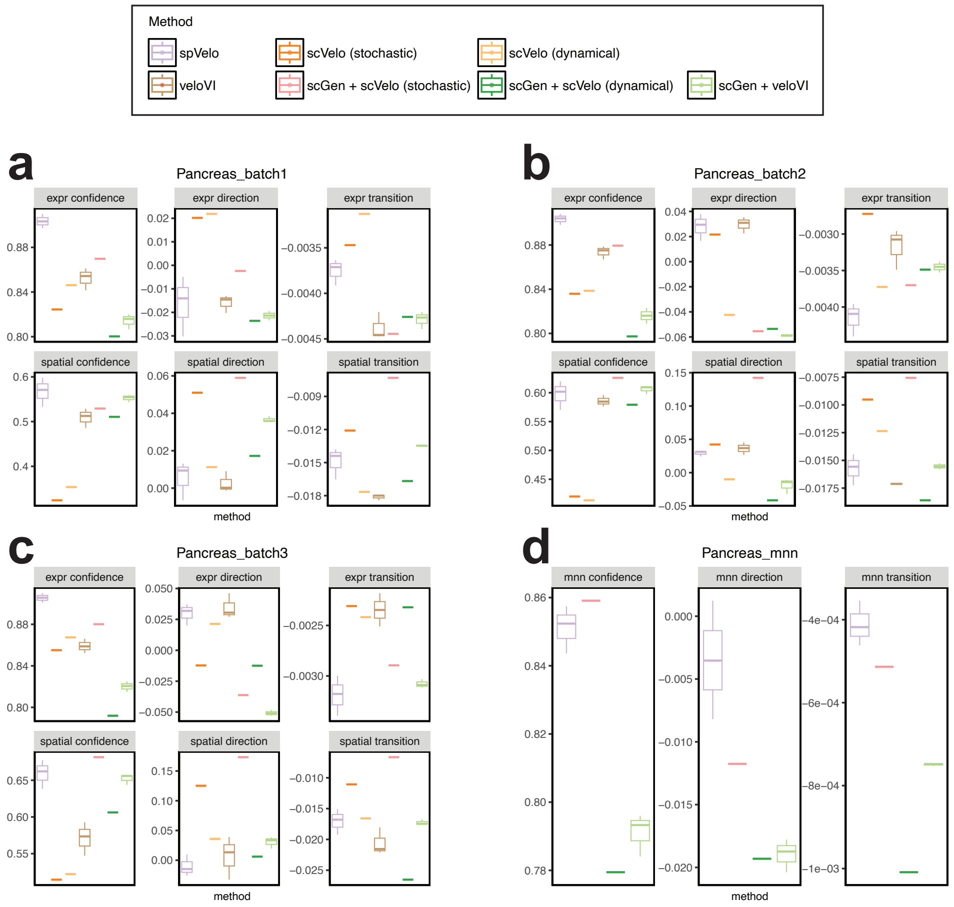

Fig. S3: Boxplots of all comparing scores in simulated pancreas dataset. (a-c) Perbatch scores from batch 1 to batch 3 of simulated pancreas dataset. (d) MNN scores of simulated pancreas dataset.

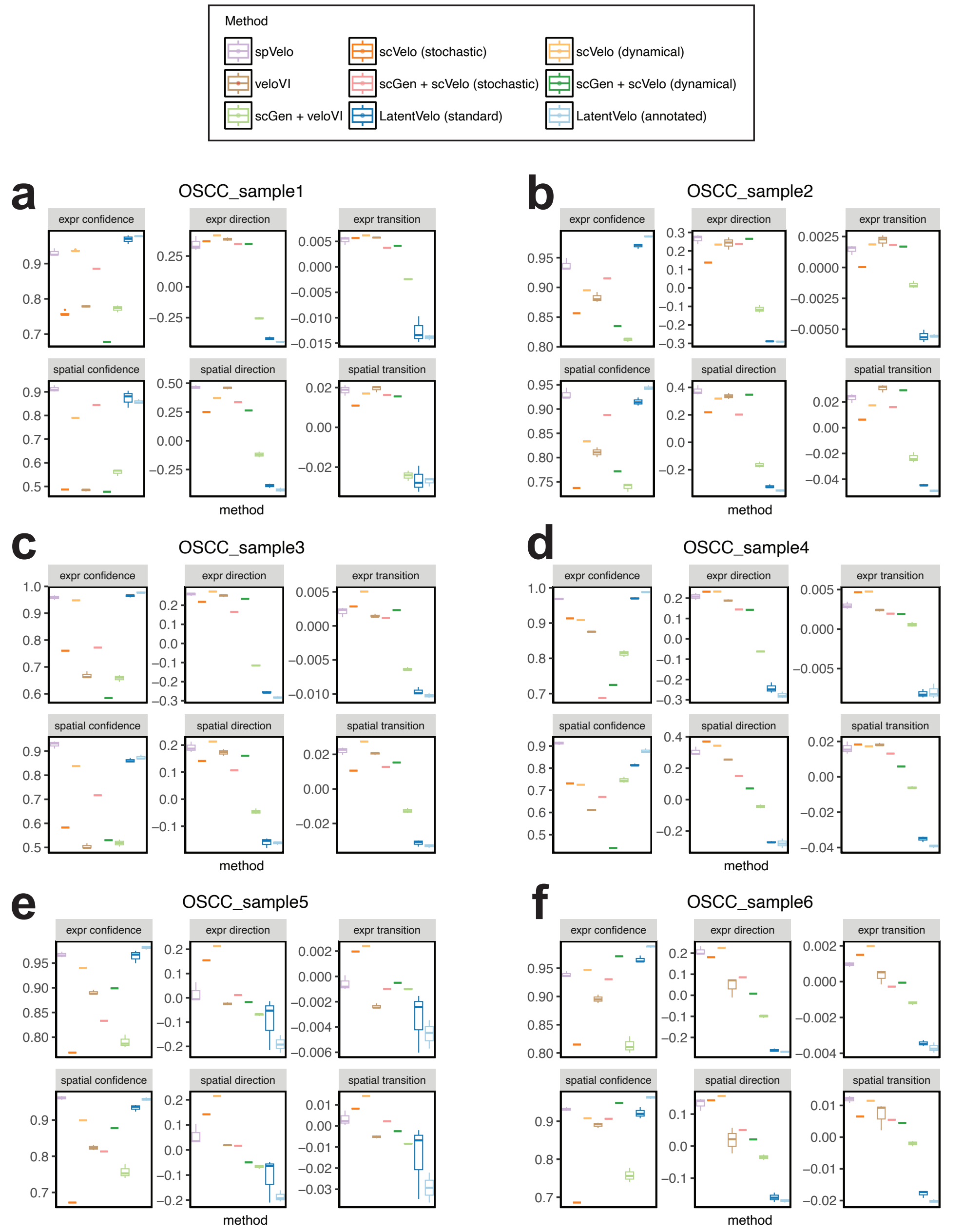

Fig. S4: Boxplots of all comparing scores in OSCC dataset. (a-f) Perbatch scores from sample 1 to sample 6 of OSCC dataset.

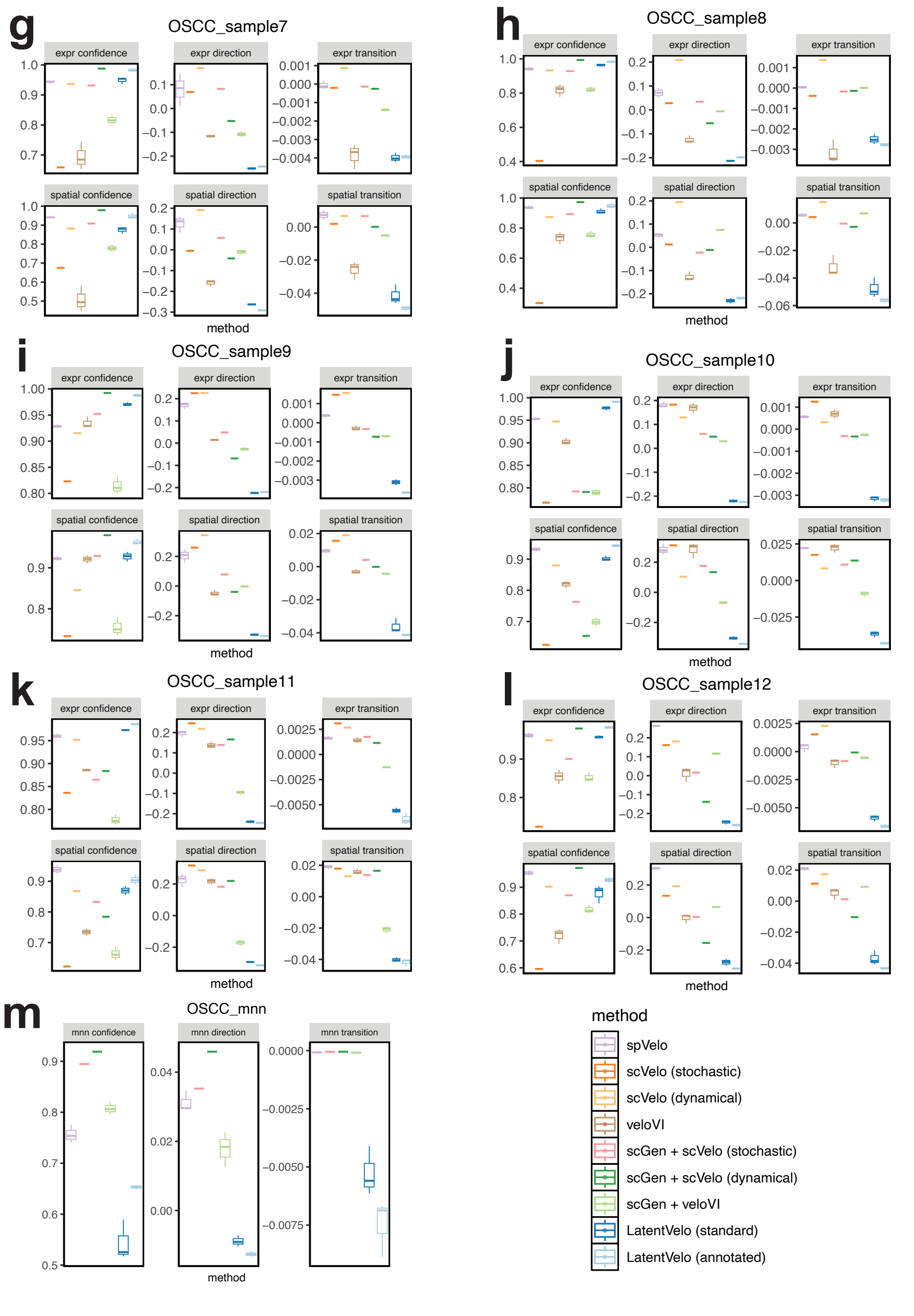

Fig. S4: **(continue)** Boxplots of all comparing scores in OSCC dataset. (g-l) Perbatch scores from sample 7 to sample 12 of OSCC dataset. (m) MNN scores of OSCC dataset.

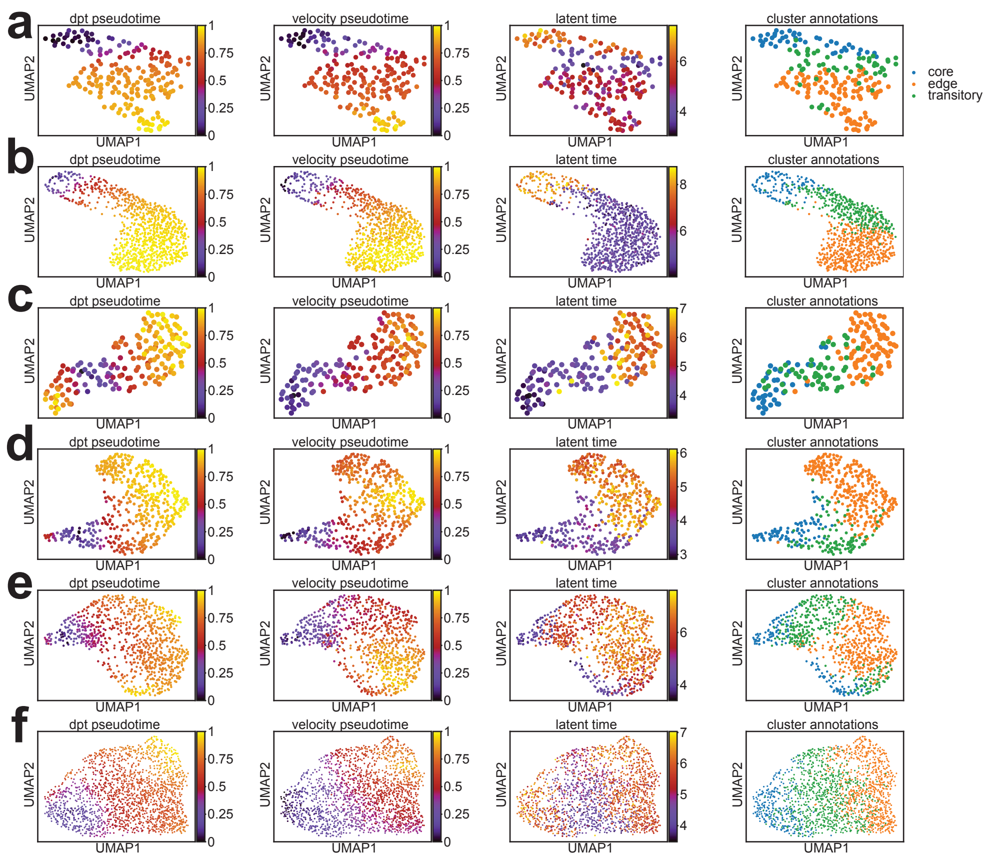

Fig. S5: Comparison of latent time inferred by spVelo with DPT and velocity pseudotime. (a-f) Scatter plots from sample 1 to sample 6.

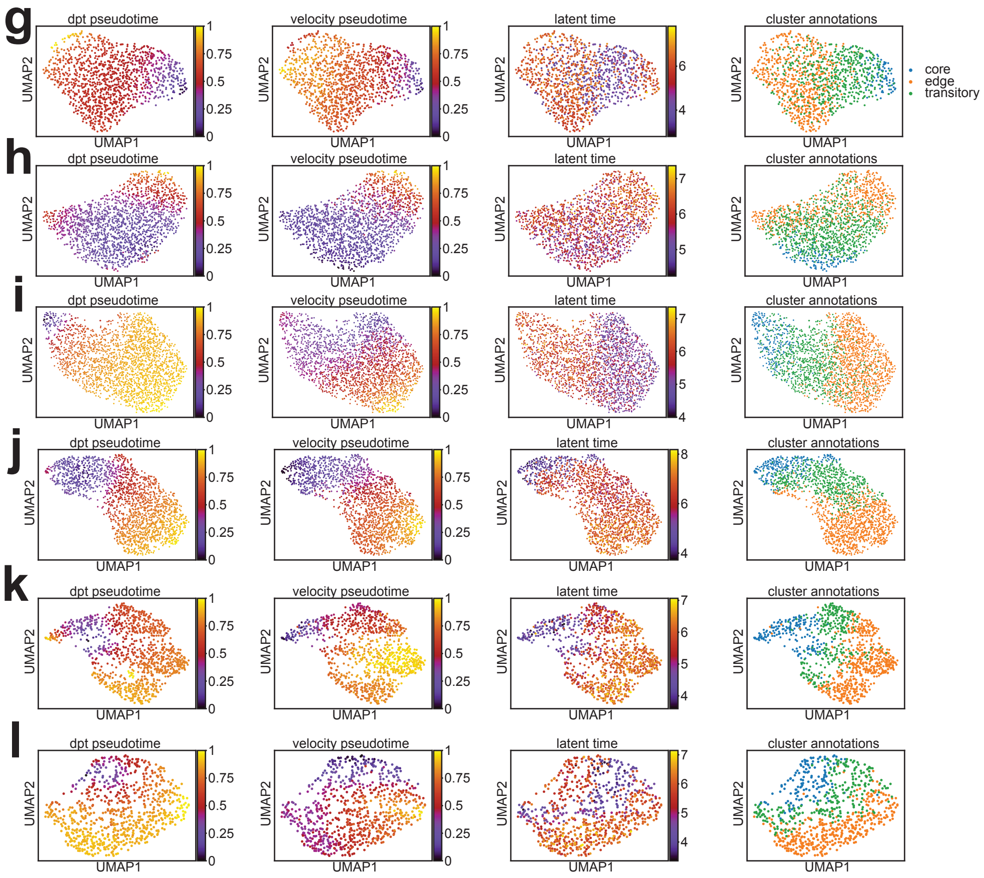

Fig. S5: **(continue)** Comparison of latent time inferred by spVelo with DPT and velocity pseudo-time. (g-l) Scatter plots from sample 7 to sample 12.

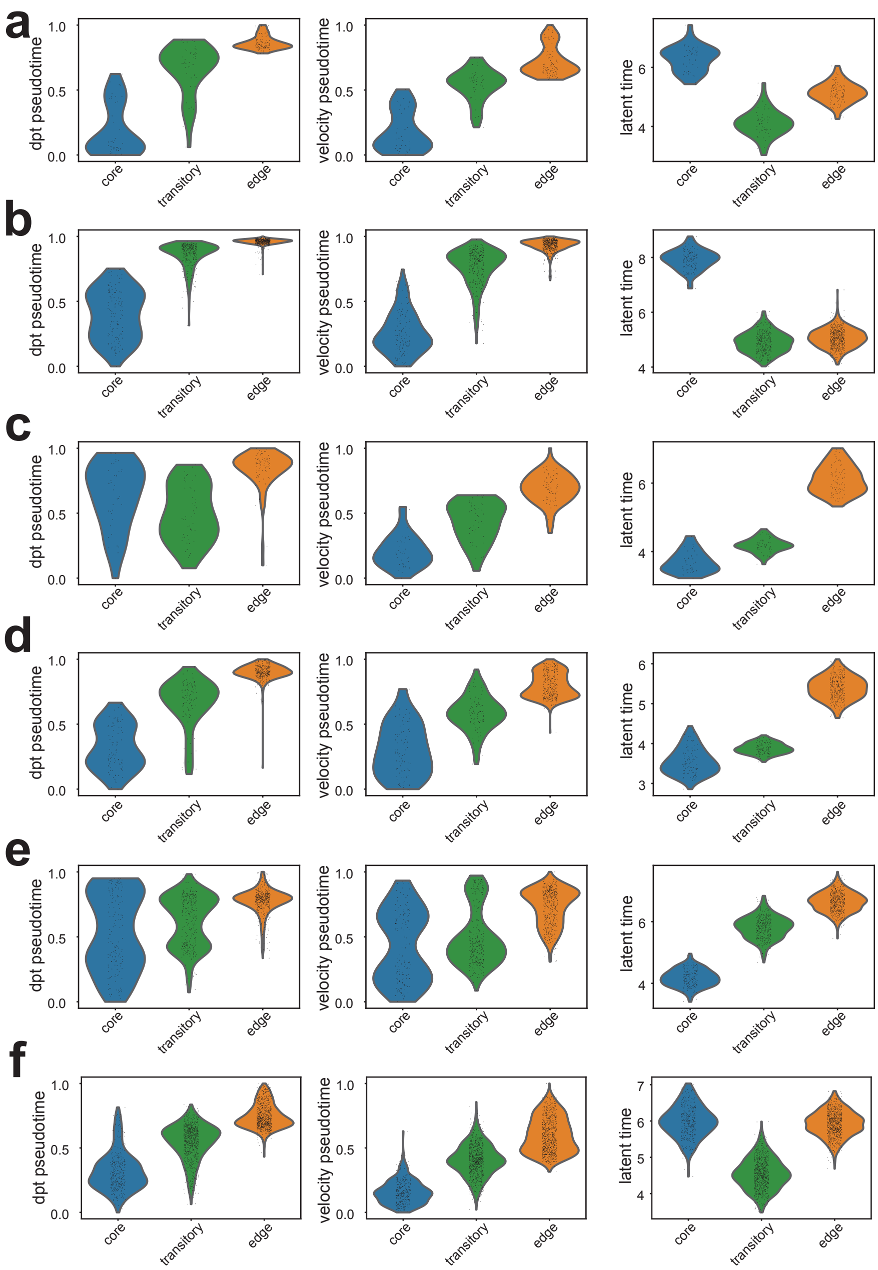

Fig. S5: **(continue)** Comparison of latent time inferred by spVelo with DPT and velocity pseudo-time. (a-f) Violin plots from sample 1 to sample 6.

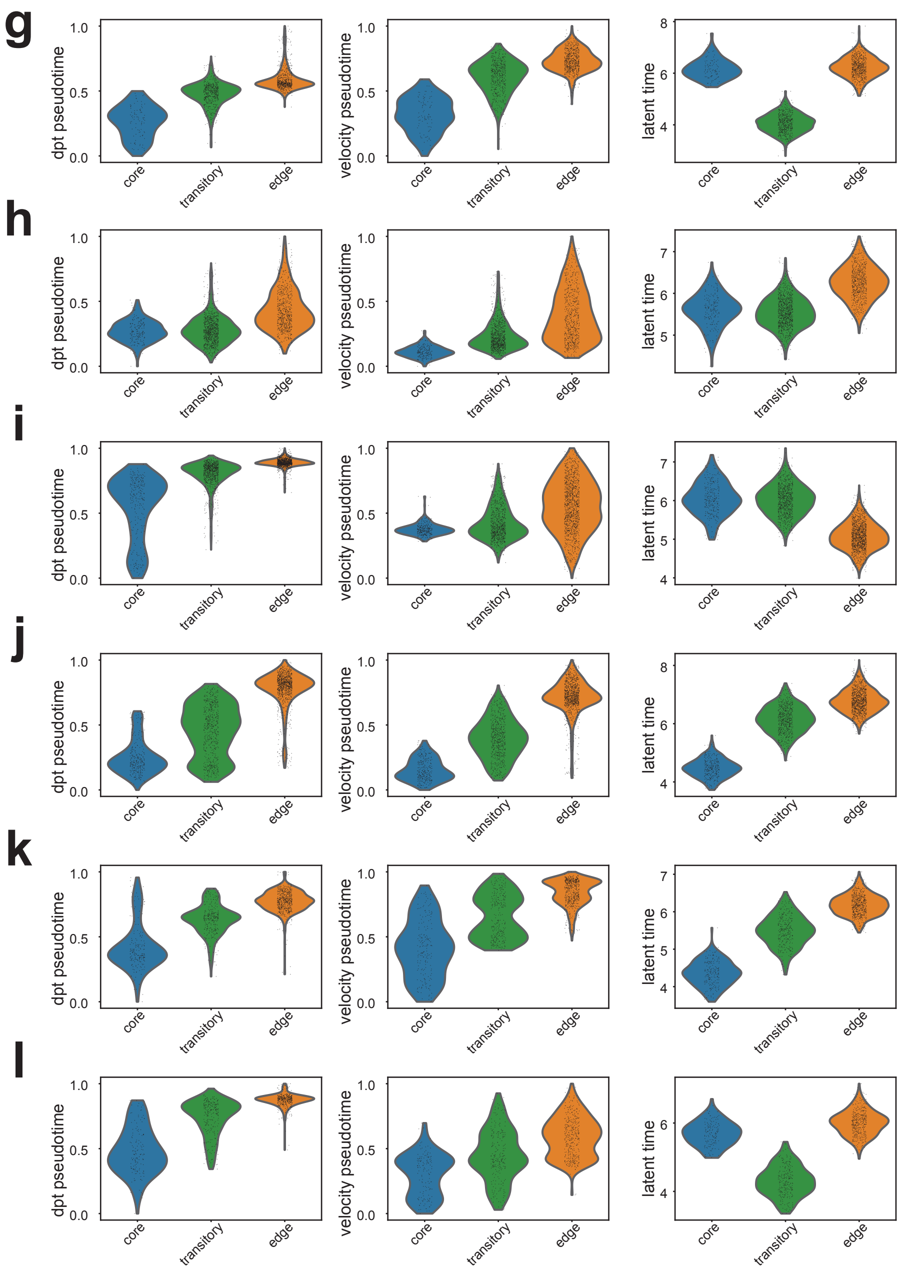

Fig. S5: **(continue)** Comparison of latent time inferred by spVelo with DPT and velocity pseudo-time. (g-l) Violin plots from sample 7 to sample 12.

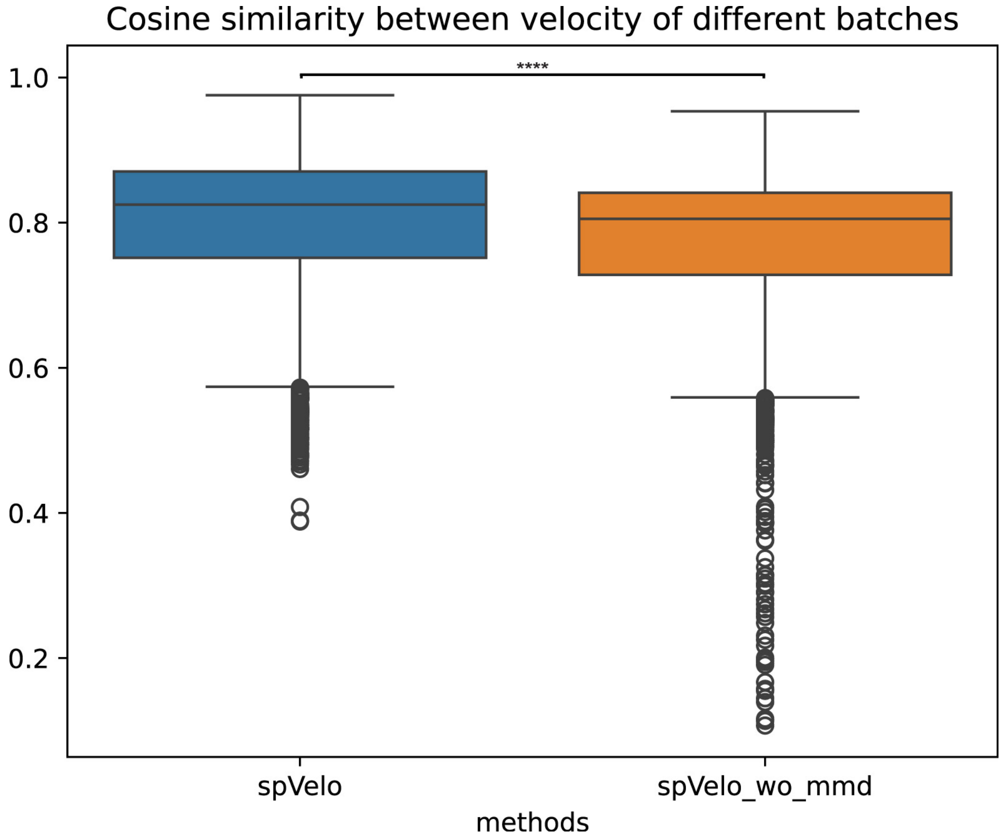

Fig. S6: Comparison of velocity coherence score between the original spVelo (spVelo) and spVelo without MMD penalty (spVelo_wo_mmd).

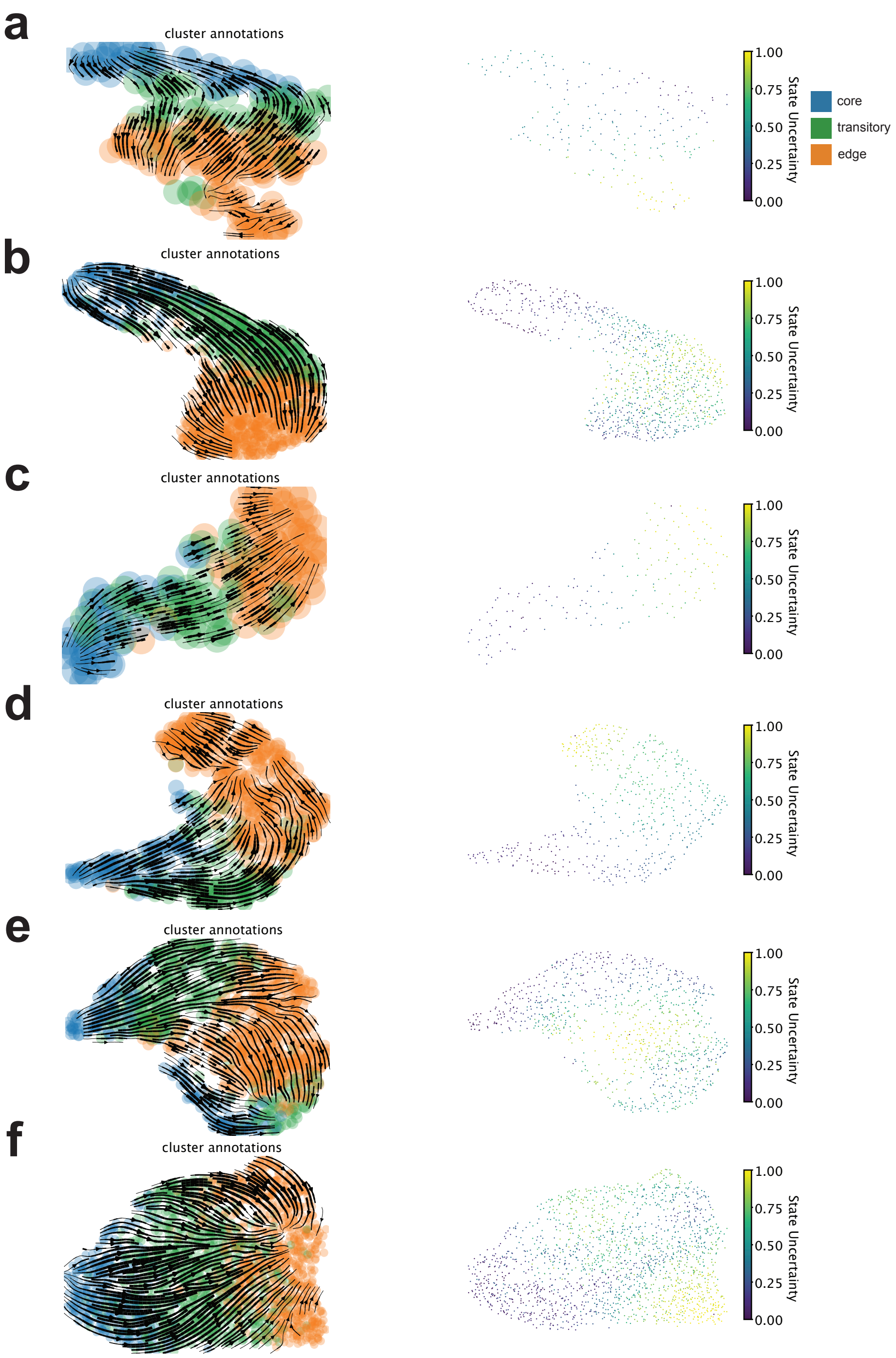

Fig. S7: Trajectory plots and uncertainty scatter plots of spVelo on OSCC dataset. (a-f) Trajectory and uncertainty plots on UMAP embedding from sample 1 to sample 6.

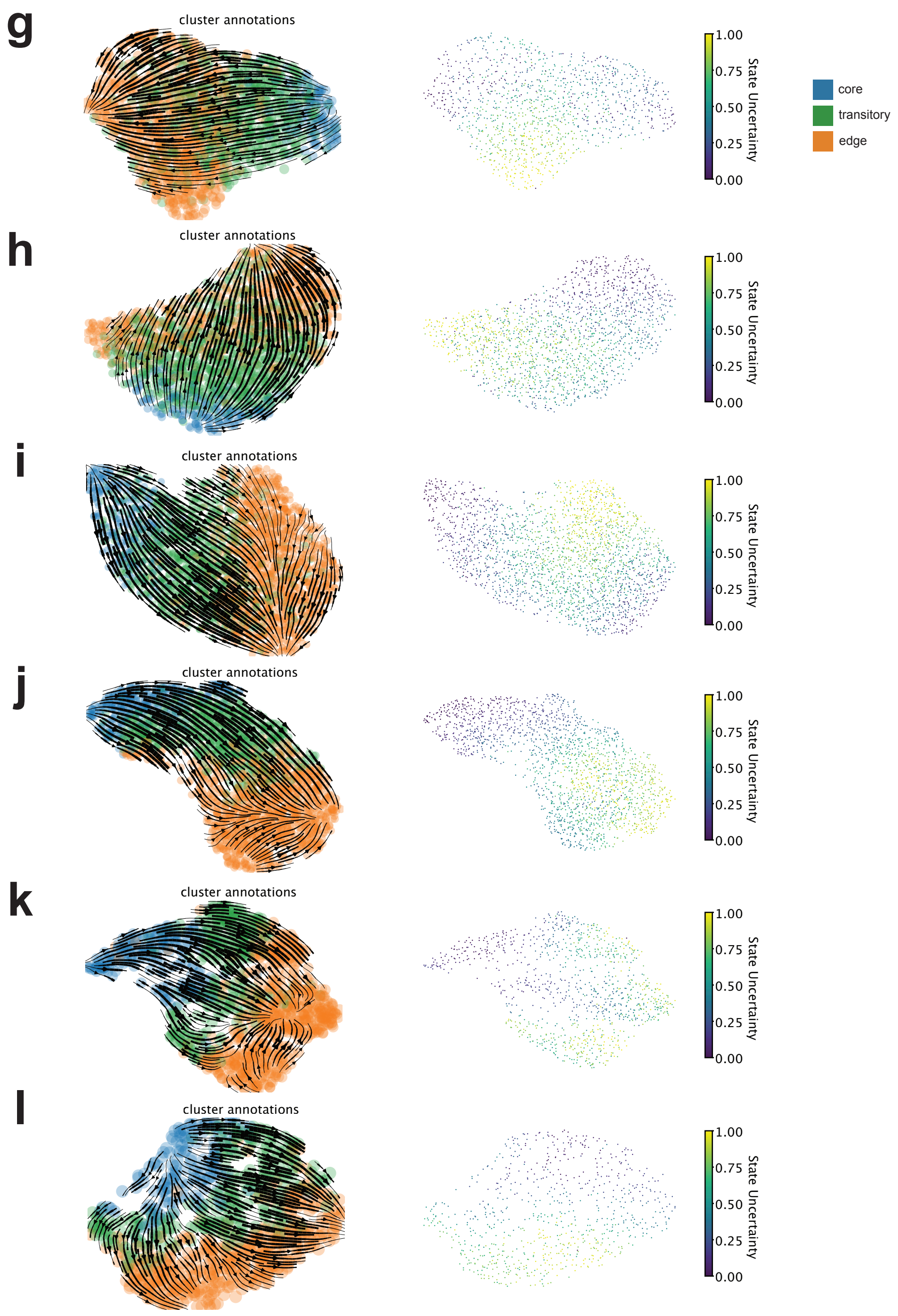

Fig. S7: **(continue)** Trajectory plots and uncertainty scatter plots of spVelo on OSCC dataset. (g-l) Trajectory plots and uncertainty on UMAP embedding from sample 7 to sample 12.

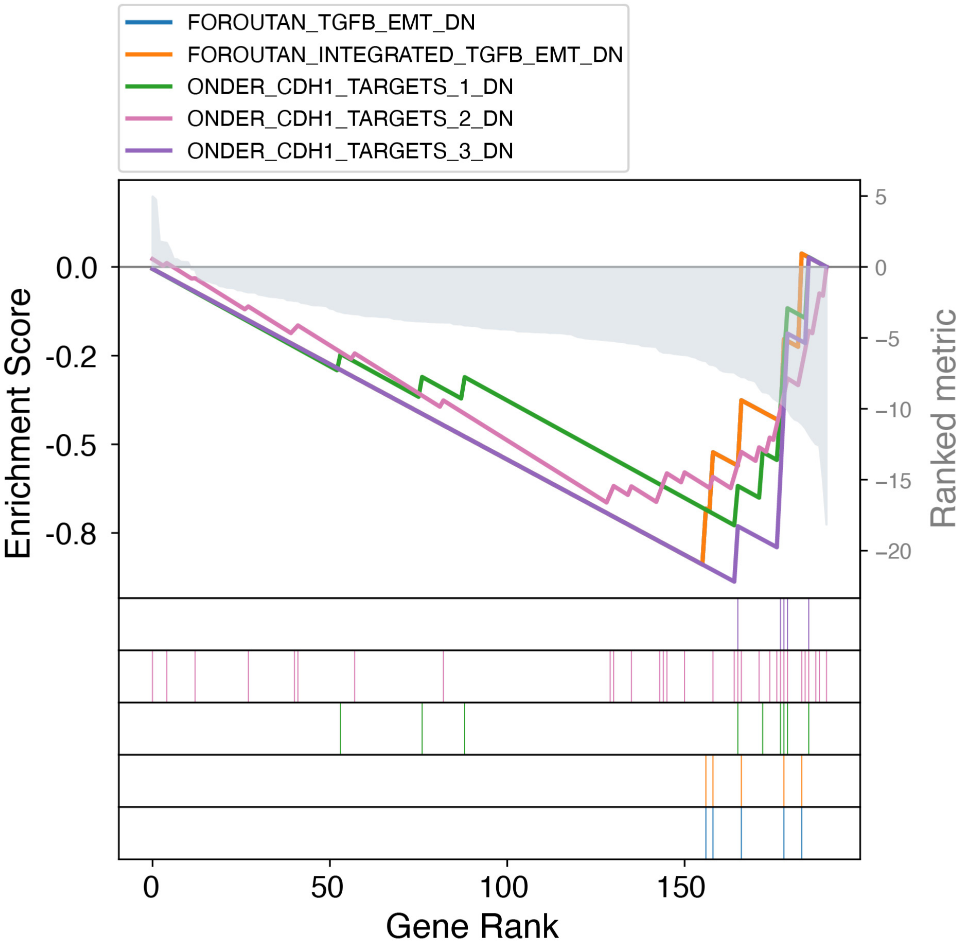

Fig. S8: Gene set enrichment analysis using prerank. Genes are ranked by Wilcoxon test statistic from differentially expression test between high-uncertainty cells and low-uncertainty cells.

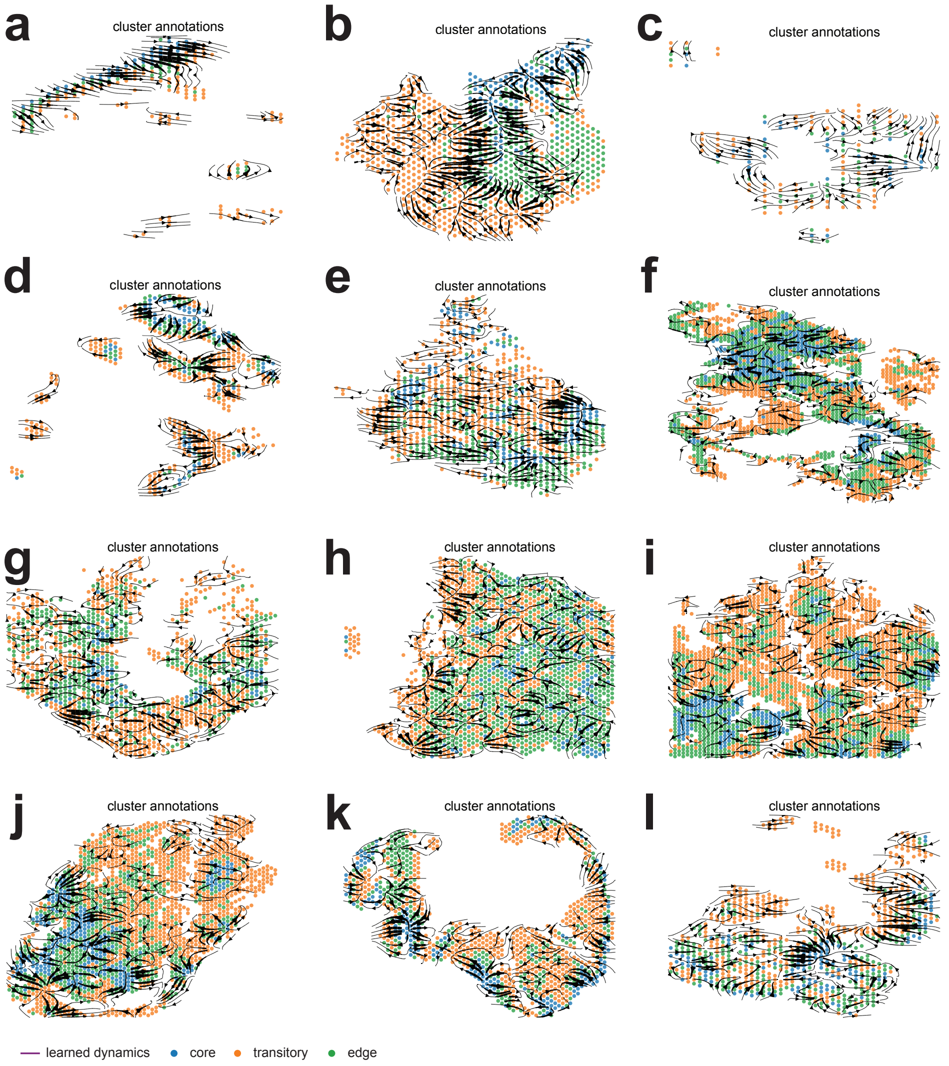

Fig. S9: Trajectory plots of spVelo on the spatial coordinate of OSCC dataset. (a-l) Trajectory plots and uncertainty on the spatial coordinate from sample 1 to sample 12.

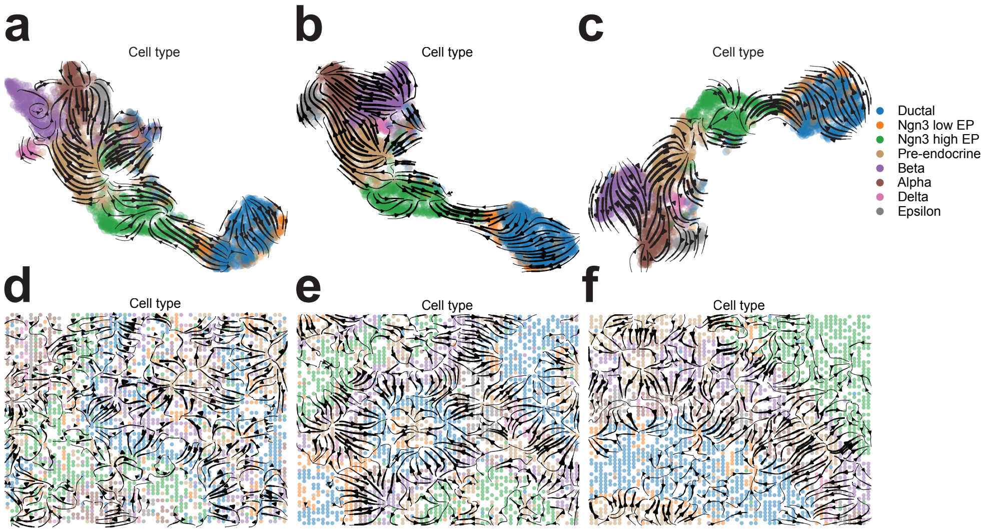

Fig. S10: Trajectory plots of spVelo on UMAP and spatial coordinate of simulated pancreas dataset. (a-c) Trajectory plots on UMAP embedding from batch 1 to batch 3. (d-f) Trajectory plots on spatial coordinate from batch 1 to batch 3.

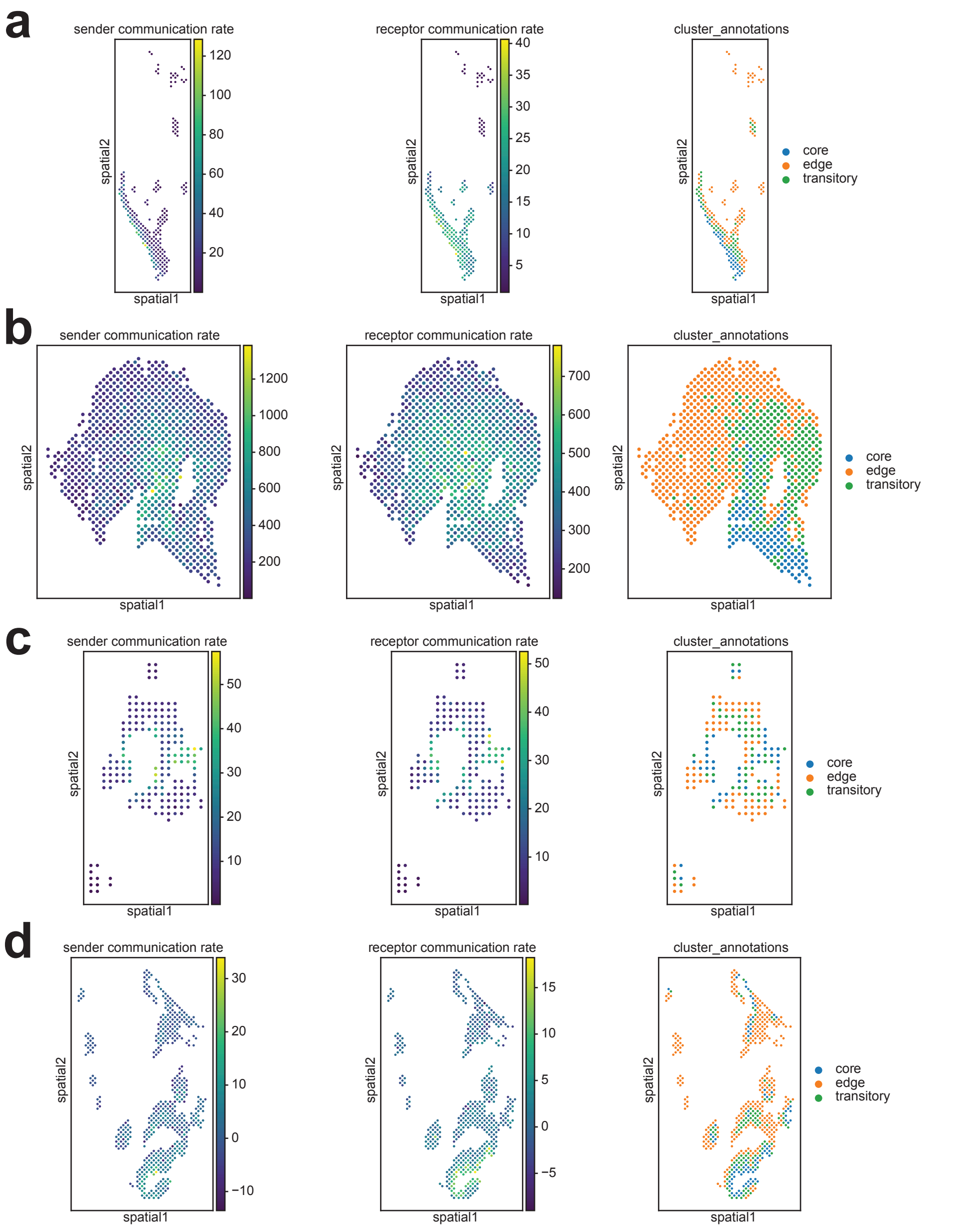

Fig. S11: Temporal cell-cell communications inference results of OSCC dataset. (a-d) Temporal cell-cell communications inference results of sample 1 to sample 4.

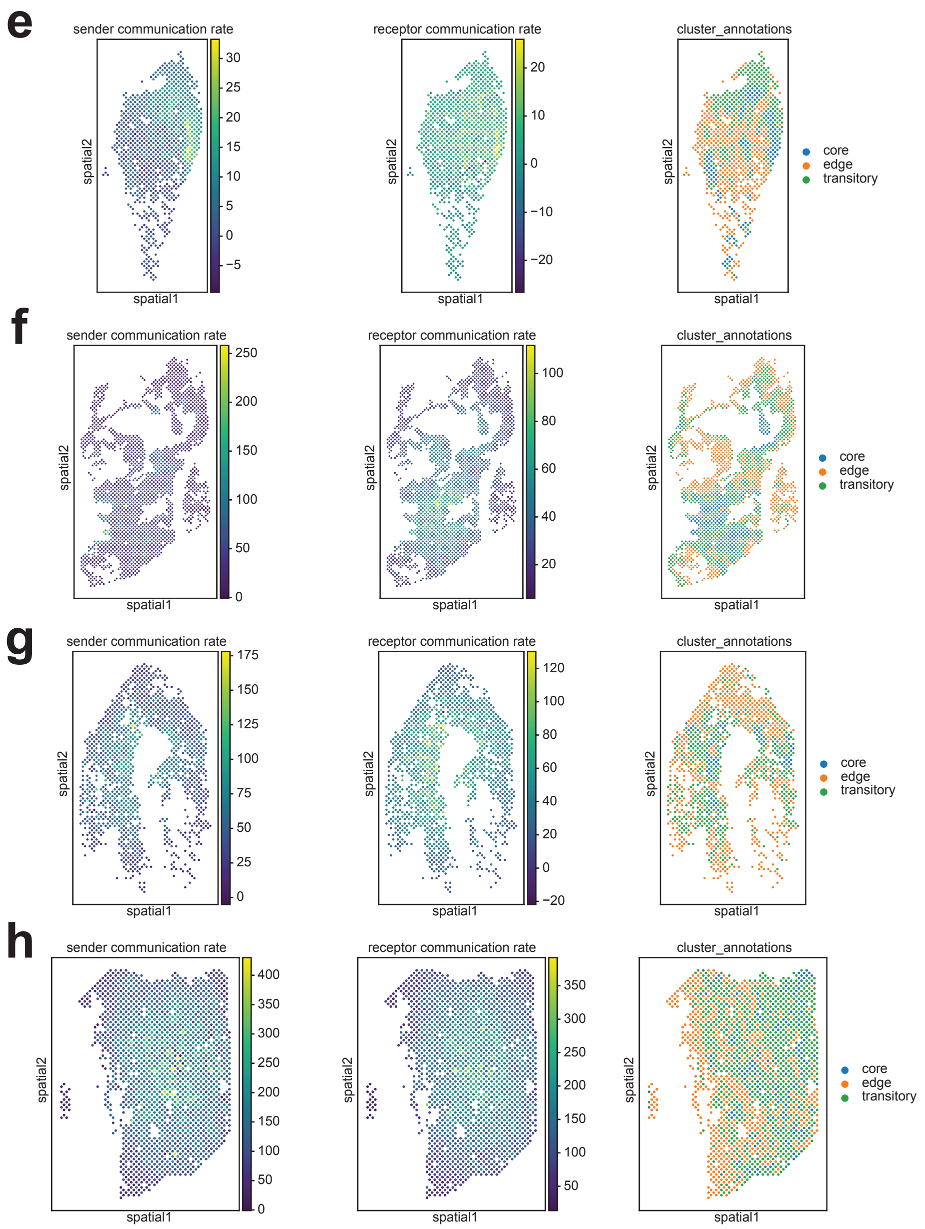

Fig. S11: **(continue)** Temporal cell-cell communications inference results of OSCC dataset. (e-h) Temporal cell-cell communications inference results of sample 5 to sample 8.

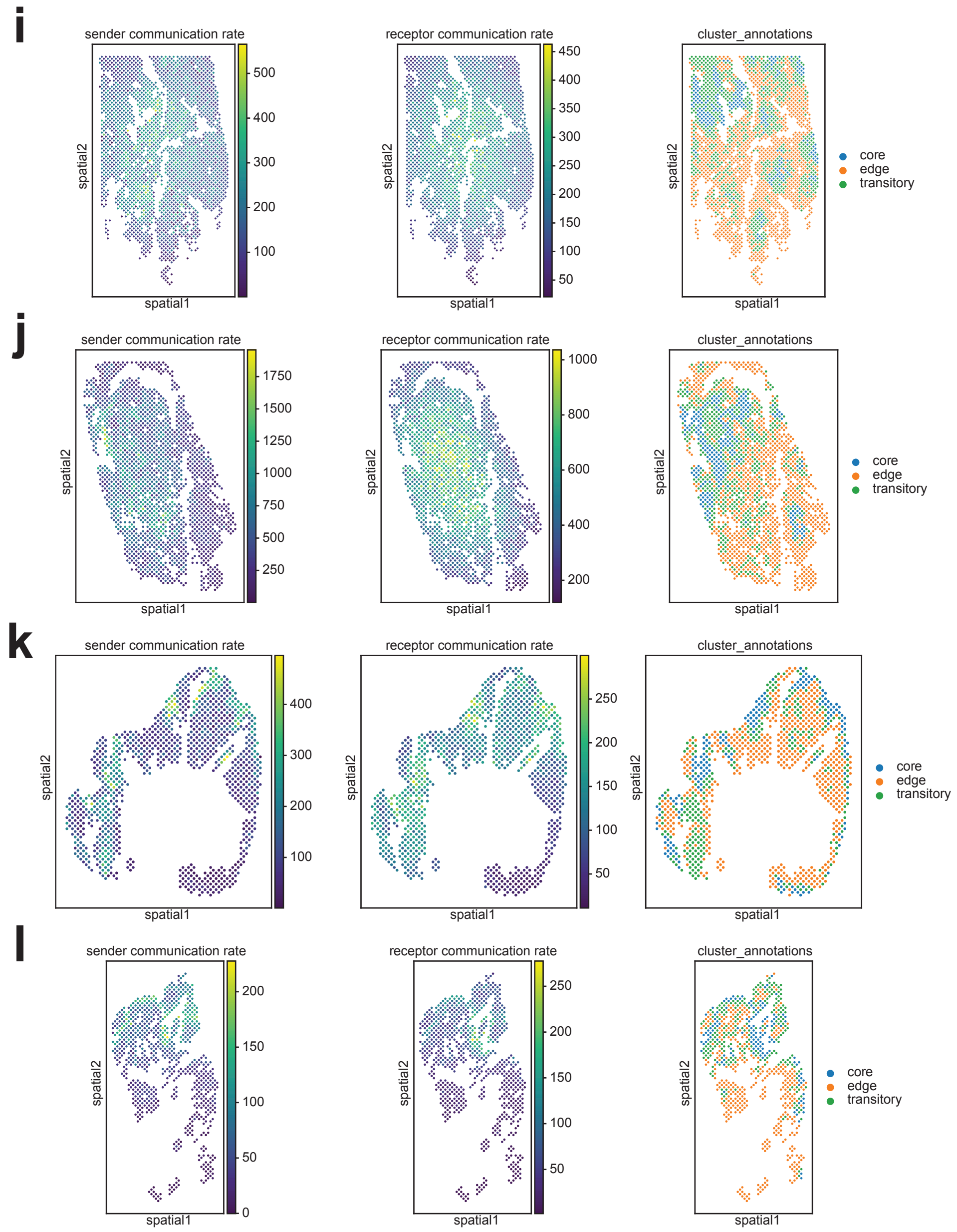

Fig. S11: **(continue)** Temporal cell-cell communications inference results of OSCC dataset. (i-l) Temporal cell-cell communications inference results of sample 9 to sample 12.

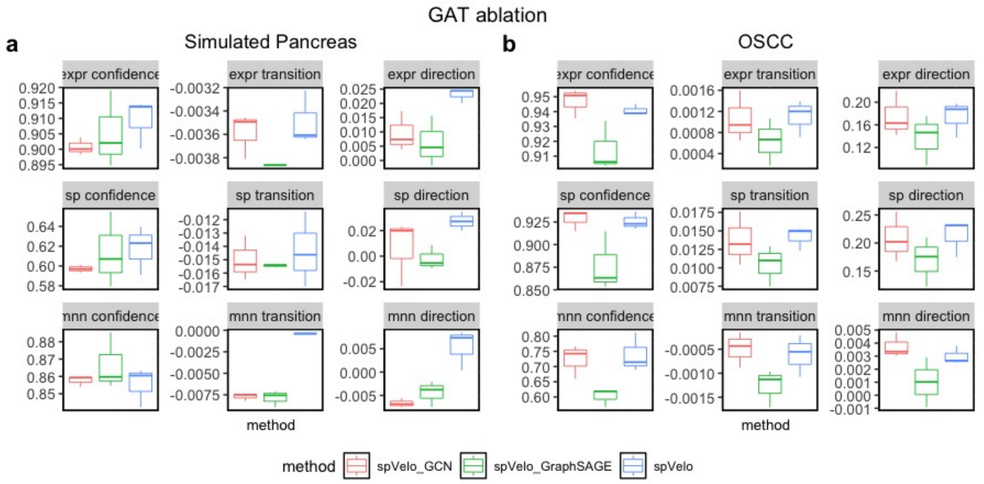

Fig. S12: Boxplots comparing the original spVelo with GAT module (spVelo), spVelo with GCN module (spVelo_GCN) and spVelo with GraphSAGE module (spVelo_GraphSAGE). (a) Comparison in the simulated Pancreas dataset. (b) Comparison in the OSCC dataset.

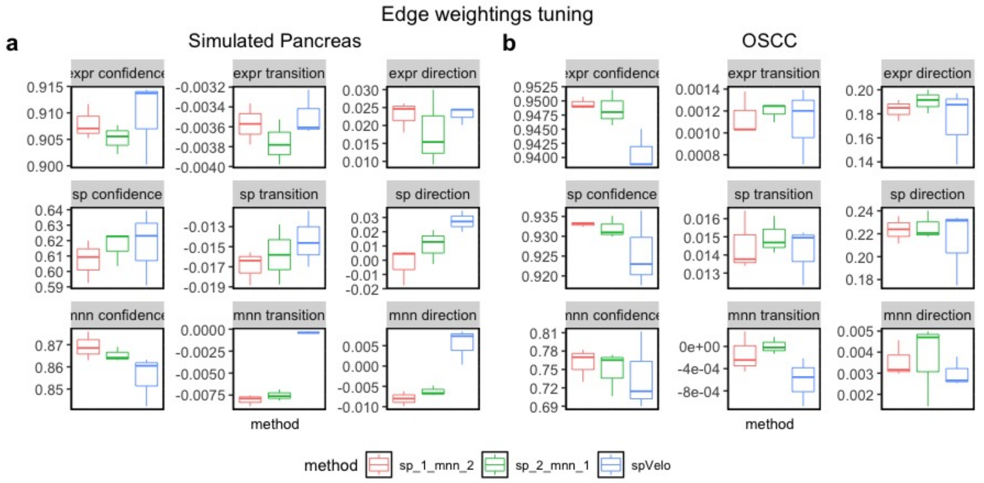

Fig. S13: Boxplots comparing the original spVelo with same weights for spatial and mnn edges(spVelo), spVelo with spatial_weight:mnn_weight=1:2 (sp_1_mnn_2) and spVelo with spatial_weight:mnn_weight=2:1 (sp_2_mnn_1). (a) Comparison in the simulated Pancreas dataset. (b) Comparison in the OSCC dataset.

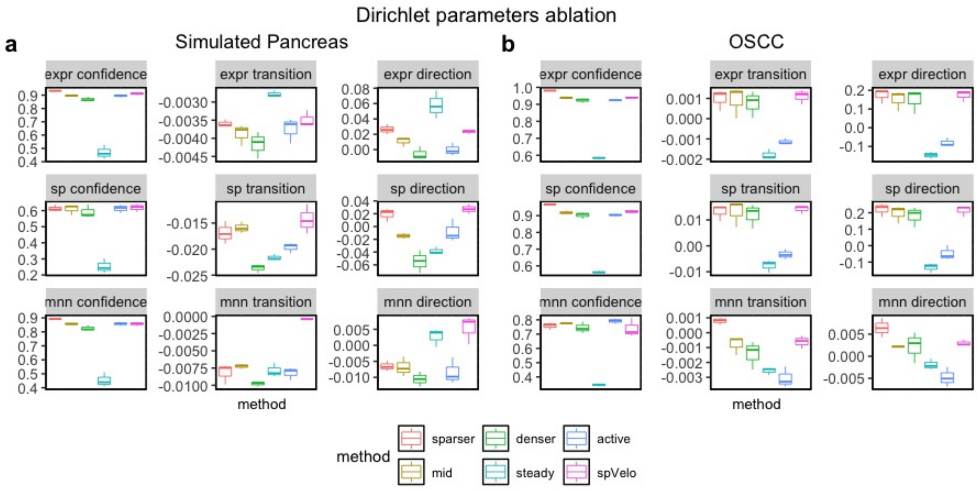

Fig. S14: Boxplots comparing different parameters of Dirichlet distribution. (a) Comparison in the simulated Pancreas dataset. (b) Comparison in the OSCC dataset.

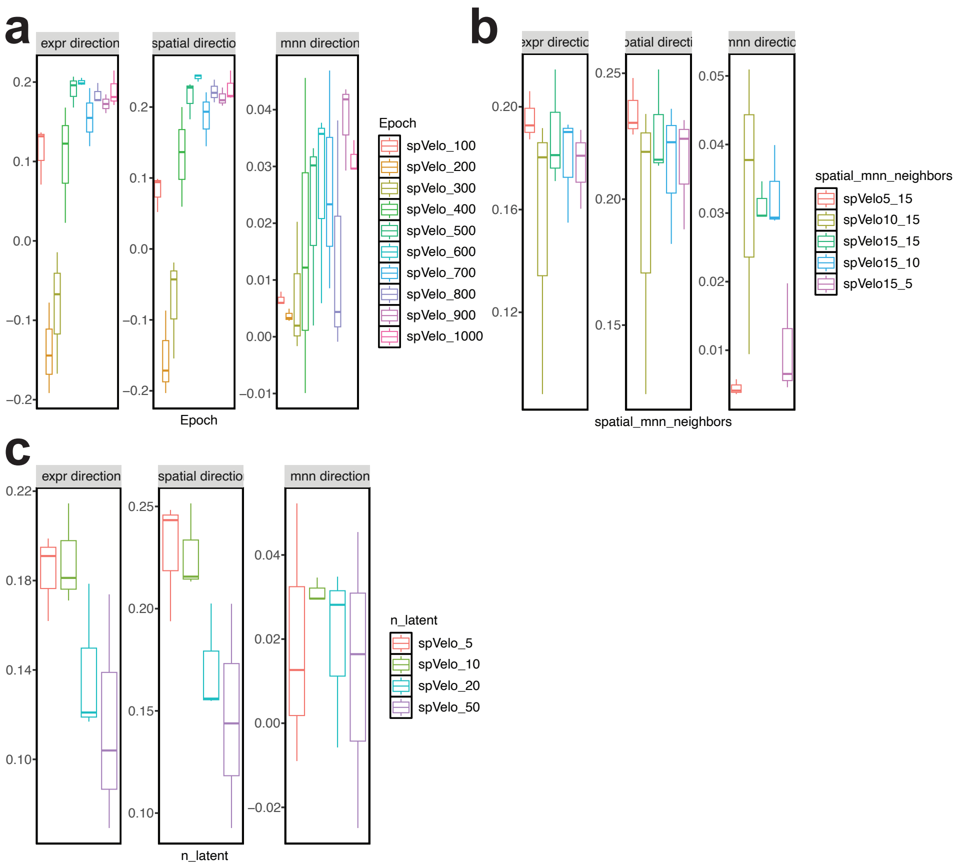

Fig. S15: Hyper-parameter tuning of spVelo on OSCC dataset. (a) The change of direction scores by adjusting the number of epochs. (b) The change of direction scores by adjusting the number of spatial and mnn neighbors. (c) The change of direction scores by adjusting the size of latent space.

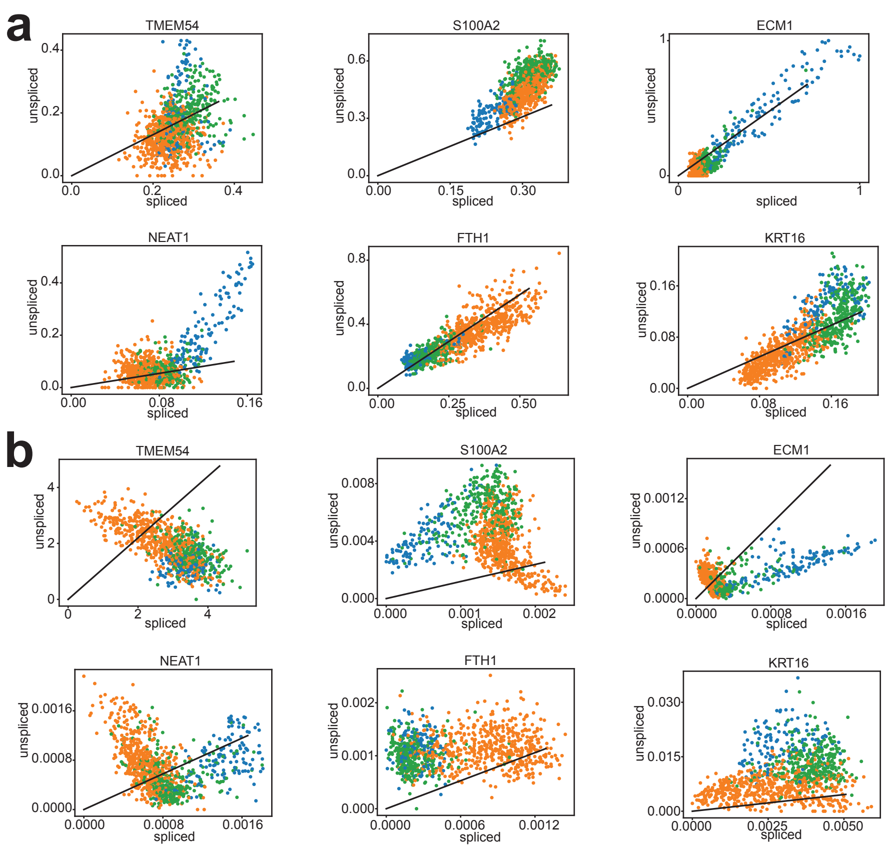

Fig. S16: scDesign3 simulation data quality. (a) Scatter plot of genes from the original dataset. (b) Scatter plot of genes from scDesign3 simulated dataset.

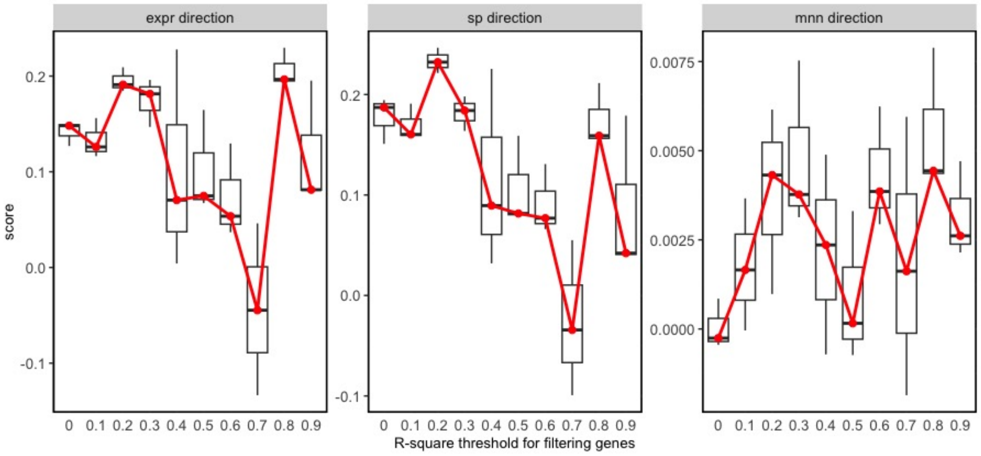

Fig. S17: Direction scores using different R-square thresholds for filtering genes.

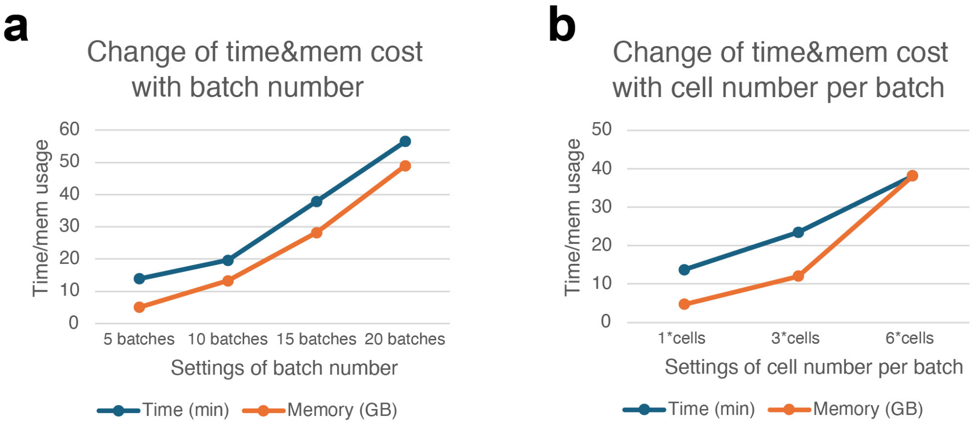

Fig. S18: Changes of running time and memory usage with batch number and cell number. (a) Changes with batch number, each batch has about 2,300 cells. (b) Changes with cell number per batch, each dataset has 3 batches.

References

[1] Qiu, X., Zhu, D.Y., Lu, Y., Yao, J., Jing, Z., Min, K.H., Cheng, M., Pan, H., Zuo, L., King, S., *et al.*: Spatiotemporal modeling of molecular holograms. Cell **187**(26), 7351–7373 (2024)

[2] Zhou, P., Bocci, F., Li, T., Nie, Q.: Spatial transition tensor of single cells. Nature Methods **21**(6), 1053–1062 (2024)

[3] Abdelaal, T., Grossouw, L.M., Pasterkamp, R.J., Lelieveldt, B.P., Reinders, M.J., Mahfouz, A.: Sirv: Spatial inference of rna velocity at the single-cell resolution. NAR genomics and bioinformatics **6**(3), 100 (2024)

[4] Maćkiewicz, A., Ratajczak, W.: Principal components analysis (pca). Computers & Geosciences **19**(3), 303–342 (1993)

[5] McInnes, L., Healy, J., Melville, J.: Umap: Uniform manifold approximation and projection for dimension reduction. arXiv preprint arXiv:1802.03426 (2018)

[6] Qiao, C., Huang, Y.: Representation learning of rna velocity reveals robust cell transitions. Proceedings of the National Academy of Sciences **118**(49), 2105859118 (2021)

[7] Farrell, S., Mani, M., Goyal, S.: Inferring single-cell transcriptomic dynamics with structured latent gene expression dynamics. Cell Reports Methods **3**(9) (2023)

[8] Qian, J., Bao, H., Shao, X., Fang, Y., Liao, J., Chen, Z., Li, C., Guo, W., Hu, Y., Li, A., *et al.*: Simulating multiple variability in spatially resolved transcriptomics with sccube. Nature Communications **15**(1), 5021 (2024)

[9] Song, D., Wang, Q., Yan, G., Liu, T., Sun, T., Li, J.J.: scdesign3 generates realistic in silico data for multimodal single-cell and spatial omics. Nature Biotechnology **42**(2), 247–252 (2024)

[10] Arora, R., Cao, C., Kumar, M., Sinha, S., Chanda, A., McNeil, R., Samuel, D., Arora, R.K., Matthews, T.W., Chandarana, S., *et al.*: Spatial transcriptomics reveals distinct and conserved tumor core and edge architectures that predict survival and targeted therapy response. Nature Communications **14**(1), 5029 (2023)

[11] Bastidas-Ponce, A., Tritschler, S., Dony, L., Scheibner, K., Tarquis-Medina, M., Salinno, C., Schirge, S., Burtscher, I., Böttcher, A., Theis, F.J., *et al.*: Comprehensive single cell mrna profiling reveals a detailed roadmap for pancreatic endocrinogenesis. Development **146**(12), 173849 (2019)
